# Post-heparin plasma lipase activities in patients with severe hypertriglyceridemia treated with evinacumab

**DOI:** 10.1016/j.jlr.2026.101020

**Published:** 2026-03-12

**Authors:** Cecilia Vitali, Poulabi Banerjee, Robert Pordy, Peter Ehmann, Robert S. Rosenson, Daniel J. Rader

**Affiliations:** 1 Department of Medicine, Perelman School of Medicine, University of Pennsylvania, Philadelphia, PA, USA; 2 Regeneron Pharmaceuticals, Inc., Tarrytown, NY, USA; 3 Metabolism and Lipids Program, Mount Sinai Fuster Heart Hospital, Icahn School of Medicine at Mount Sinai, New York, NY, USA; 4 Department of Genetics, Perelman School of Medicine, University of Pennsylvania, Philadelphia, PA, USA

**Keywords:** Clinical trials, genomics, lipase, lipoproteins, triglycerides

## Abstract

Patients with severe hypertriglyceridemia (sHTG) have variable lipoprotein lipase (LPL) activity levels that may influence therapeutic response. This exploratory analysis investigated post-heparin triglyceride lipase and phospholipase activities in three cohorts of patients with sHTG who received evinacumab (angiopoietin-like 3 inhibitor) for 12 or 24 weeks during a phase 2 trial: cohort 1, familial chylomicronemia syndrome with bi-allelic loss-of-function (LOF) LPL pathway mutations; cohort 2, multifactorial chylomicronemia syndrome (MCS) with heterozygous LOF LPL pathway mutations; and cohort 3, MCS without LPL pathway mutations. Post-heparin plasma samples were obtained at baseline and at week 24 (end of the treatment period). Triglyceride lipase activities (LPL and hepatic lipase [HL]) were measured using both a colorimetric and a scintillation assays. Phospholipase activities (HL and endothelial lipase [EL]) were measured using a colorimetric assay. Baseline post-heparin LPL triglyceride lipase activity was lowest in cohort 1; treatment with evinacumab for 12 or 24 weeks did not alter activity at week 24 versus baseline across cohorts using the colorimetric assay. Non-HL triglyceride lipase activity (mostly LPL) assessed using the scintillation assay showed a significant increase in cohort 1 at 24 weeks versus baseline (*P* = 0.04). Neither HL nor EL phospholipase activities differed among cohorts or changed with evinacumab treatment. High intra- and inter-patient variability in lipase activity was observed with all methods. Post-heparin LPL triglyceride lipase activity was lower in patients with sHTG with bi-allelic LPL pathway mutations and increased in that group with evinacumab. The high variability in lipase activities observed via differing methods supports the need for more robust assays.

## Human genes

*ABCA1,* adenosine triphosphate binding cassette subfamily A member 1;

*ABCG5,* adenosine triphosphate binding cassette subfamily G member 5;

*ABCG8,* adenosine triphosphate binding cassette subfamily G member 8;

*ANGPTL3,* angiopoietin-like 3;

*ANGPTL4,* angiopoietin-like 4;

*ANGPTL8,* angiopoietin-like 8;

*APOA1,* apolipoprotein A1;

*APOA4,* apolipoprotein A4;

*APOA5,* apolipoprotein A5;

*APOC2,* apolipoprotein C2;

*APOC3,* apolipoprotein C3;

*APOD,* apolipoprotein D;

*BTN2A1,* butyrophilin subfamily 2 member A1;

*COL18A1,* collagen type XVIII alpha 1 chain;

*CREB3L3,* cyclic adenosine monophosphate responsive element binding protein 3 like 3;

*GALNT2,* polypeptide N-acetylgalactosaminyltransferase 2;

*GCKR,* glucokinase regulator;

*GPD1,* glycerol-3-phosphate dehydrogenase 1;

*GPIHBP1,* glycosylphosphatidylinositol anchored high density lipoprotein binding protein 1;

*LIPI,* lipase I;

*LMF1,* lipase maturation factor 1;

*LPL,* lipoprotein lipase;

*LRP8,* low-density lipoprotein receptor related protein 8;

*MLXIPL,* MLX interacting protein like;

*PCSK7,* proprotein convertase subtilisin/kexin type 7;

*TIMD4,* T cell immunoglobulin and mucin domain containing 4;

*TRIB1,* tribbles pseudokinase 1;

*USF1,* upstream transcription factor 1;

Severe hypertriglyceridemia (sHTG) is a condition characterized by very high fasting blood triglyceride levels, conventionally defined as ≥500 mg/dl (>5.64 mmol/l) (1, 2) (https://www.accessdata.fda.gov/drugsatfda_docs/label/2019/202057s035lbl.pdf). In the United States, more than 3 million adults are estimated to be affected by sHTG (3). sHTG is a known risk factor not only for atherosclerotic cardiovascular disease but also for acute pancreatitis (AP), for which it is estimated to cause or contribute to ∼10% of cases (4).

Triglycerides are synthesized and secreted by the liver in very-low-density lipoprotein (VLDL) or are transported within chylomicrons after lipid absorption in the intestine and circulate in triglyceride-rich lipoproteins (TRLs) (5, 6, 7). The triglycerides in TRLs are mostly hydrolyzed by lipoprotein lipase (LPL) in muscle (skeletal and cardiac) and adipose tissue (8). LPL is synthesized by parenchymal cells in these tissues and transported to the capillary lumen by glycosylphosphatidylinositol-anchored high-density lipoprotein binding protein 1 (GPIHBP1), which also docks LPL on the endothelial surface (5, 6, 8).

In the capillary lumen, LPL hydrolysis of triglycerides within chylomicrons and VLDL releases free fatty acids and monoglycerides for tissue uptake, resulting in chylomicron remnants and intermediate-density lipoprotein, which are cleared by the liver (9, 10). LPL activity plays a critical role in lipid homeostasis and is therefore highly regulated at the transcriptional, post-transcriptional, translational, and post-translational levels (11). LPL activity is promoted by the lipoprotein-associated apolipoprotein C2 (APOC2) and apolipoprotein A5 (APOA5) proteins, and is inhibited by several circulating proteins, including apolipoprotein C3 (APOC3), angiopoietin-like 3 (ANGPTL3), and angiopoietin-like 4 (ANGPTL4) (10).

Patients with sHTG have chylomicronemia, which is characterized by the intermittent or persistent presence of chylomicrons in plasma under fasting conditions (12). Chylomicronemia is most commonly a polygenic disorder, known as multifactorial chylomicronemia syndrome (MCS), caused by multiple triglyceride-elevating genetic variants that can be affected by lifestyle, comorbidities, and medications (13). In contrast, familial chylomicronemia syndrome (FCS) is a rare monogenic disorder caused by loss-of-function (LOF) variants in *LPL* or other genes of the LPL pathway, including *APOA5*, *APOC2*, *GPIHBP1*, and *LMF1* (13, 14).

Two other extracellular lipases that influence lipoprotein metabolism are hepatic lipase (HL) and endothelial lipase (EL) (15). Both enzymes have triglyceride lipase and phospholipase activities that contribute to the catabolism of TRLs (15).

ANGPTL3 is an endogenous inhibitor of both LPL and EL but not HL (16, 17, 18). Evinacumab, a monoclonal antibody against ANGPTL3, reduces plasma triglyceride levels as well as low-density lipoprotein cholesterol levels (18, 19). Evinacumab is approved in the European Union/European Economic Area and Canada as an adjunct to diet and other lipid-lowering therapies (LLTs) for the treatment of homozygous familial hypercholesterolemia (HoFH) in patients aged ≥5 years (https://www.ema.europa.eu/en/documents/product-information/evkeeza-epar-product-information_en.pdf; https://pdf.hres.ca/dpd_pm/00072602.PDF). In the United States, evinacumab is approved as an adjunct to other LLTs for the treatment of HoFH in patients aged ≥5 years (https://www.regeneron.com/sites/default/files/Evkeeza_PI.pdf). Evinacumab also has age-agnostic approval in Japan for the treatment of HoFH, and is approved in the UK as an adjunct to diet and other LLTs for the treatment of HoFH in patients aged ≥5 years (https://www.ultragenyx.com/wp-content/uploads/2024/01/Ultragenyx_Press_Release_Evkeeza_solution_345mg_received_approval_for_manufacturing_and_marketing_in_Japan_01182024.pdf; https://mhraproducts4853.blob.core.windows.net/docs/d82840a338e8ba5a930feba9e16ae38b5ca9b8de).

Evinacumab is hypothesized to provide clinical benefit to patients with a history of AP and sHTG and LOF mutations in *LPL* genes; the reduction in serum triglyceride levels from baseline is expected to reduce the risk of future AP episodes. Here, we share the findings from an exploratory analysis investigating the association between post-heparin LPL activity and genetic variants in patients with sHTG who participated in a phase 2 trial with evinacumab (NCT03452228) (20).

## Materials and methods

This study was conducted in accordance with ethical principles originating from the Declaration of Helsinki and in accordance with the International Conference on Harmonization/Good Clinical Practices and applicable regulatory requirements. All patients provided written informed consent. The protocol, any amendments, informed consent form, Investigator Brochure, and other relevant documents were reviewed and approved by each institution’s Institutional Review Board and/or Ethics Committees before the study was initiated.

### Phase 2 study design and treatment

The trial design and results of the phase 2 study with evinacumab used for this exploratory analysis have been previously published (20). Briefly, adults (aged 18–75 years) with sHTG (fasting serum triglycerides >500 mg/dl [>5.64 mmol/l] at screening on two separate occasions, documented medical history of fasting triglycerides ≥1,000 mg/dl [≥11.29 mmol/l]), and a history of hospitalization for acute pancreatitis were enrolled based on genotype according to the presence of LOF genetic variants in LPL pathway genes (20). A full list of the study inclusion and exclusion criteria are provided in the Supplemental methods. All patients were subsequently exome sequenced and analyzed by the Regeneron Genetics Center (RGC; Regeneron Pharmaceuticals, Inc.). The effect of evinacumab was evaluated in three cohorts of patients: cohort 1, patients with FCS with bi-allelic LOF LPL pathway mutations (n = 17); cohort 2, patients with MCS with heterozygous LOF LPL pathway mutations (n = 15); and cohort 3, patients with MCS without LPL pathway mutations (n = 19). This study did not include a control group with normal/healthy individuals. The study comprised a screening phase, a 4-week single-blind placebo run-in, a 12-week double-blind treatment period (DBTP), a 12-week single-blind treatment period (SBTP), and a 20-week off-drug observation phase (Supplemental Fig. S1). Patients in each cohort were randomized 2:1 to receive either intravenous evinacumab 15 mg/kg every 4 weeks or matching placebo for the 12-week DBTP. After completion of the DBTP, all patients entered the SBTP and received intravenous evinacumab 15 mg/kg every 4 weeks for 12 weeks, including the placebo-treated patients, who were switched to evinacumab. Stable doses of other lipid-modifying therapies, such as fibrates, statins, omega-3s, niacin, and lomitapide, were allowed during the study. Samples obtained at baseline and week 24 were analyzed, by which point all patients had received evinacumab for at least 12 weeks.

Triglyceride lipase activity was measured via colorimetric (LPL and HL activity) and scintillation assays (HL and non-HL), and phospholipase activities (HL and EL) were measured using a colorimetric assay; both assay methods are described below. Samples for lipase activity determination were collected at baseline (after confirmation of patient eligibility during the screening period) and at the end of the treatment period. Fasting plasma was collected from patients after they received an intravenous bolus of heparin 60 IU/kg.

We also analyzed the correlation between the change from baseline to week 24 in triglycerides and the change in lipase activity over the same time period.

### Exome sequencing and analysis

Genomic DNA was extracted from peripheral blood samples and submitted for whole exome sequencing at the RGC. Further details of the exome sequencing and analysis are provided in the Supplemental methods. Individuals in this study were screened for variants in a list of 28 genes compiled for their reported associations with triglyceride or lipid levels. Screened genes were: *ABCA1, ABCG5, ABCG8, ANGPTL3, ANGPTL4, ANGPTL8, APOA1, APOA4, APOA5, APOC2, APOC3, APOD, BTN2A1, COL18A1, CREB3L3, GALNT2, GCKR, GPD1, GPIHBP1, LIPI, LMF1, LPL, LRP8, MLXIPL, PCSK7, USF1, TIMD4,* and *TRIB1.*

### Determination of triglyceride lipase activity: colorimetric assays

Post-heparin LPL activity was measured using the assay developed by Imamura and colleagues (21). In brief, LPL and HL present in the post-heparin plasma sample act on a natural substrate, diglyceride, to release monoglyceride. The released monoglyceride is then hydrolyzed by monoglyceride lipase, which has high specificity for monoglycerides, into glycerol and free fatty acid. Glycerol kinase acts on the glycerol to produce glycerol-3-phosphate, which in turn is acted upon by glycerol-3-phosphate oxidase to generate hydrogen peroxide. The hydrogen peroxide is then quantified by the enzymatic activity of peroxidase, which converts the hydrogen peroxide, 4-aminoantipyrine, and TOOS N-ethyl-N-(2-hydroxy-3-sulfopropyl)-m-toluidine (TOOS) into a quinoneimine dye. The rate of formation of the dye, measured as an increase in absorbance at 550 nm, is proportional to the lipase activity. LPL activity requires the presence of APOC2, whereas HL activity does not require APOC2. Therefore, LPL and HL activities are measured with and without the presence of APOC2, respectively, in a two-channel autoanalyzer; LPL activity can thus be calculated as the difference between the two assays.

### Determination of phospholipase activity: colorimetric assays

Post-heparin total and HL phospholipase activities were measured according to the method of Miksztowicz and colleagues (22), with minor adaptations. EL phospholipase activity was determined as the difference between total and HL enzyme activities. Briefly, 20 μl of post-heparin plasma (1:10 dilution) was added to a 96-well plate. For the determination of total phospholipase activity, samples were preincubated with water, whereas for the determination of HL phospholipase activity they were preincubated with 1 M NaCl (Thermo Fisher Scientific, Waltham, MA, USA). At the end of the incubation period, each sample received 75 μl of either 1-decanoylthio-1-deoxy-2-decanoyl-sn-glycero-3-phosphoryl ethylene glycol (ThioPEG; Avanti Polar Lipids, Alabaster, AL, USA) substrate or blank substrate. The ThioPEG and blank substrate were prepared as follows. ThioPEG in chloroform (Avanti Polar Lipids) was dried under nitrogen flow to remove solvent. A pH 8.3 and 7 mM Triton X-100 buffer containing 100 mM HEPES (4-(2-hydroxyethyl)-1-piperazineethanesulfonic acid) was added to the vial and sonicated. A solution of 271 mM 5,5′-dithio-bis(2-nitrobenzoic acid) (DTNB; Sigma-Aldrich, Burlington, MA, USA) in dimethyl sulfoxide was prepared by vortexing, and was added to the ThioPEG emulsion immediately before use. The final ThioPEG and DTNB concentration in the emulsion was 4.3 mM. The blank substrate was prepared in the same manner as the ThioPEG substrate but did not contain ThioPEG. Absorbance at 412 nm was recorded over time using a microplate reader (PowerWave XS2, BioTek Instruments, Inc; 37°C read frequency of every 39 s for 40 min). Phospholipase activity was calculated based on the rate of increase in absorbance in the linear range of the curve (10–25 min) after subtracting the rate of increase of the corresponding blank. Enzyme activity was calculated as previously described (22), and was expressed as μmol of fatty acid released per hour per ml of post-heparin plasma. An internal control (the post-heparin plasma pool) was included in each plate and was used to control for inter-plate variability.

### Determination of triglyceride lipase activity: scintillation assays

Total lipase activity and HL activity were measured according to the method of McCoy and colleagues (23), with minor modifications, as detailed in this section. Lipase activities were assessed in post-heparin plasma samples taken at baseline and at 24 weeks after initiation of the study, which coincided with the completion of the 12–24-week treatment period with evinacumab (Supplemental Fig. S1). Non-HL triglyceride lipase activity was determined as the difference between total and HL enzyme activities and mainly represents LPL activity. (23).

Briefly, triglyceride lipase activity was measured using a glycerol-stabilized emulsion of triolein, phosphatidylcholine, and ^3^H-triolein as the substrate and post-heparin plasma as the enzyme source. Concentrated emulsion was prepared as follows: 1.12 mCi ^3^H-triolein (PerkinElmer, Inc), 300 mg triolein (MilliporeSigma, Burlington, MA, USA), and 18 mg egg phosphatidylcholine (MilliporeSigma) in organic solvents were added to a glass tube. The solvent was subsequently evaporated under nitrogen flow, and 5 ml of glycerol (Thermo Fisher Scientific) was added to the tube and sonicated with a Branson Sonifier (Thermo Fisher Scientific). The concentrated emulsion was allowed to clear overnight and was used to prepare the reaction mixtures.

The reaction mixture for the total lipase activity assay contained, in a total volume of 300 μl, 5 μl post-heparin plasma (enzyme source), 0.05 M tris(hydroxymethyl)aminomethane hydrochloride pH 8.0 (Thermo Fisher Scientific), 0.75% bovine serum albumin (Millipore Sigma), 3.4 mM triolein and ∼250 μM phosphatidylcholine (from the concentrated emulsion), 0.15 M NaCl, and 1.1 mg/ml purified APOC2 (Millipore Sigma).

The reaction mixture used for the HL activity assay had a similar composition but did not contain purified APOC2; the final concentration of NaCl was 1 M. The reaction mixture was incubated for 1 h at 37°C. Reactions were extracted by adding 3.25 ml of a mixture of methanol:chloroform:heptane (Thermo Fisher Scientific) at the ratio of 1.41:1.25:1.00, followed by 1.05 ml of potassium carbonate/potassium tetraborate/potassium hydroxide/ethylenedinitrilotetraacetic acid disodium salt dihydrate buffer pH 10 (Thermo Fisher Scientific). The liberated fatty acids were quantitated by scintillation counting of a 0.5 ml aliquot of the aqueous phase. Enzyme activity was calculated as previously described (23), and expressed as nmol of fatty acid released per minute per ml of post-heparin plasma. Samples were tested in technical duplicates. A control sample (post-heparin plasma pool) and a blank sample were included in each batch of analyzed samples, and these were used to control for inter-batch variability and non-specific hydrolysis, respectively.

### Statistical analysis

Descriptive statistics are reported as mean (standard deviation) or median (interquartile range [IQR]) for continuous data and count (percentage) for categorical data. Differences between cohorts at baseline were tested using one-way analysis of variance with post-hoc Tukey’s Honestly Significant Difference tests to evaluate pairwise comparisons. Changes from baseline (at week 24) were evaluated within each cohort using paired *t*-tests. Pearson correlation coefficients (*r*) are reported for correlations between two continuous measurements. A type 1 error rate (α) of 0.05 was used as a threshold for all statistical tests. All statistical analysis and data visualization were performed using R version 4.2.3 (R Development Core Team).

## Results

### Patient demographics and genotyping data

Overall, 51 patients with sHTG were enrolled and treated during the DBTP study phase. Of these, 47 patients completed the DBTP and entered the SBTP. The remaining 4 (8.5%) patients discontinued from the study due to withdrawal of consent.

Baseline demographics and lipid parameters are summarized in Table 1. The mean age was 47.8 years, and 52.9% were male. At baseline, median (IQR) fasting triglycerides were 2,267.0 mg/dl (1,076.0–3,722.0; 22.6 [12.1–42.0] mmol/l). A summary of patient genotype by cohort is presented in Supplemental Table S1. Among patients in cohort 1 with FCS with bi-allelic LOF LPL pathway mutations, 47% (n = 8) had homozygous mutations, and 29.4% (n = 5) had compound heterozygous mutations. In cohort 2 (patients with MCS with heterozygous LOF LPL pathway mutations), 53.5% (n = 8) had heterozygous LPL mutations.

**Table 1:** Demographics and baseline characteristics of patients (safety analysis set; DBTP)

Parameter	Cohort 1 (N = 17)	Cohort 2 (N = 15)	Cohort 3 (N = 19)	Evinacumab IV 15 mg/kg Q4W (N = 51)
Age, years, mean (SD)	48.9 (11.7)	50.3 (11.4)	44.8 (10.3)	47.8 (11.1)
Sex, male, n (%)	10 (58.8)	8 (53.3)	9 (47.4)	27 (52.9)
Race, n (%)				
White	15 (88.2)	12 (80.0)	14 (73.7)	41 (80.4)
Black or African American	0	1 (6.7)	0	1 (2.0)
Asian	1 (5.9)	1 (6.7)	4 (21.1)	6 (11.8)
Other	1 (5.9)	1 (6.7)	1 (5.3)	3 (5.9)
Ethnicity, n (%)				
Hispanic or Latino	2 (11.8)	2 (13.3)	2 (10.5)	6 (11.8)
Not Hispanic or Latino	15 (88.2)	13 (86.7)	17 (89.5)	45 (88.2)
BMI, kg/m^2^, mean (SD)	26.7 (4.7)	30.1 (5.0)	29.2 (4.4)	28.6 (4.8)
Baseline lipids, mg/dl, median (IQR)				
Fasting triglycerides^a^	3,223.7 (2,723.7–3,931.3)	1,238.0 (943.7–3,022.7)	1,549.0 (1,069.3–2,189.5)	2,267.0 (1,076.0–3,722.0)
Total cholesterol	381.0 (284.0–412.0)	193.0 (180.5–240.5)	275.0 (196.5–371.5)	277.0 (197.0–392.5)
Non-HDL-C	359.0 (263.0–398.0)	169.0 (146.5–221.0)	264.0 (170.5–340.0)	263.0 (171.0–374.5)
LDL-C^b^	16.0 (13.0–26.0)	50.0 (26.5–62.0)	43.0 (28.5–66.0)	31.0 (17.0–59.0)
HDL-C	18.0 (16.0–20.0)	22.0 (17.5–24.0)	21.0 (17.5–26.5)	20.0 (16.5–24.0)
APOB	64.0 (56.0–90.0)	90.0 (77.0–101.0)	99.0 (79.0–125.5)	90.0 (64.0–107.0)

APOB, apolipoprotein B; BMI, body mass index; DBTP, double-blind treatment period; HDL-C, high-density lipoprotein cholesterol; IV, intravenous; LDL-C, low-density lipoprotein cholesterol; Q4W, every 4 weeks; SD, standard deviation.

a Lab SI unit conversation for triglycerides; divide the value in mg/dl by 88.57.

b LDL-C was determined by ultracentrifugation.

### Baseline LPL triglyceride lipase activity across the three cohorts selected based on LPL genetics

**Fig. 1:**
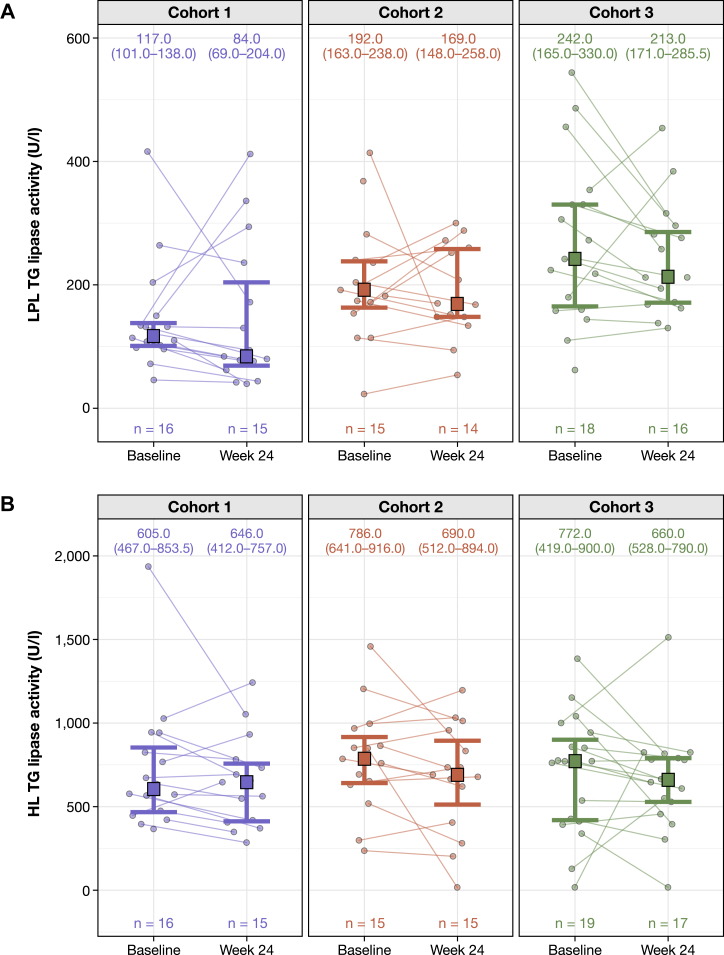
Assessment of (A) LPL and (B) HL triglyceride lipase activity via colorimetric assays shown for individuals and grouped according to cohort^a^. ^a^Bold lines indicate median (IQR) values. HL, hepatic lipase; IQR, interquartile range; LPL, lipoprotein lipase; TG, triglyceride.

Baseline post-heparin LPL triglyceride lipase activity assessed using the colorimetric assay was lowest in cohort 1 (bi-allelic), intermediate in cohort 2 (mono-allelic), and highest in cohort 3 (no mutations). The difference in baseline LPL triglyceride lipase activity was significant between cohorts 1 and 3 (*P* = 0.007) (Fig. 1A; Supplemental Tables S2 and S3). LPL activity showed high intra-patient variability at differing time points as well as high variability between individuals (Fig. 1A). The coefficients of variation (CVs; SD divided by the mean) ranged from 37.2% to 81.8%.

**Fig. 2:**
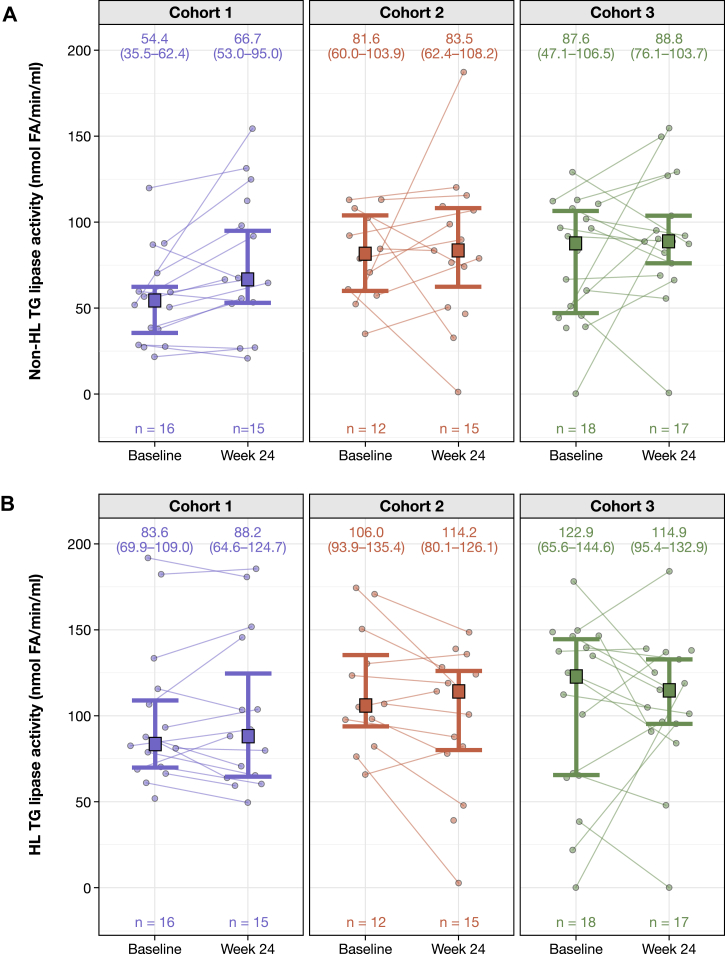
Assessment of (A) non-HL and (B) HL triglyceride lipase activity via scintillation assays shown for individuals and grouped according to cohort^a^. ^a^Bold lines indicate median (IQR) values. FA, fatty acid; HL, hepatic lipase; IQR, interquartile range; TG, triglyceride.

In contrast to LPL triglyceride lipase, no differences between cohorts were observed in HL triglyceride lipase activity at baseline for either assay type (Figs. 1B and 2B; Supplemental Tables S2–S4). CVs for HL triglyceride lipase activity ranged from 40.1% to 53.2%. Non-HL (primarily LPL) triglyceride lipase activity assessed via scintillation assays was also lowest in cohort 1, while cohorts 2 and 3 were similar; the difference in cohort 1 did not reach statistical significance (*P* = 0.05; Fig. 2A; Supplemental Tables S3 and S4). For both assays, there was high intra-cohort variability.

**Fig. 3:**
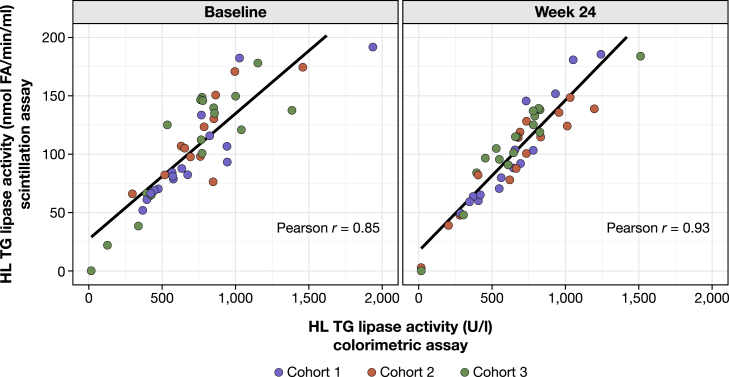
Scatterplots of HL triglyceride-lipase activity measured with scintillation and colorimetric assays at baseline and week 24. HL, hepatic lipase; TG, triglyceride.

We also evaluated whether there was a correlation between triglyceride lipase activity assessed via colorimetric and scintillation assays. To this end, we performed a linear regression analysis of HL triglyceride lipase activity values measured with colorimetric and scintillation assays (Fig. 3). At baseline and week 24, the Pearson correlation coefficients were 0.85 and 0.93, respectively.

### Triglyceride lipase activity after evinacumab treatment was assessed using the colorimetric and scintillation assays

At week 24, after at least 12 weeks of treatment with evinacumab, median LPL triglyceride activity by the colorimetric assay was not significantly different compared with baseline in all three cohorts (Fig. 1A; Supplemental Tables S2 and S3). Using the scintillation assay, the median non-HL (mostly LPL) triglyceride lipase activity in cohort 1 increased significantly from 54.4 at baseline to 66.7 nmol fatty acid (FA)/min/ml at 24 weeks (*P* = 0.04). No significant changes were observed in cohorts 2 (*P* = 0.70) and 3 (*P* = 0.29) (Fig. 2A; Supplemental Tables S3 and S4). HL activity measured by scintillation assay showed a significant increase in cohort 2 (*P* = 0.037), but not in cohorts 1 (*P* = 0.94) and 3 (*P* = 0.98) at week 24 (Fig. 2B, Supplemental Table S3 and S4). There was no significant increase in HL activity measured by the colorimetric assay across cohorts (Fig. 1A; Supplemental Tables S2 and S3). At baseline and week 24, the Pearson correlation coefficients for change in LPL triglyceride and HL activity were 0.62 and 0.54, respectively.

### Assessment of phospholipase activity via colorimetric assays

**Fig. 4:**
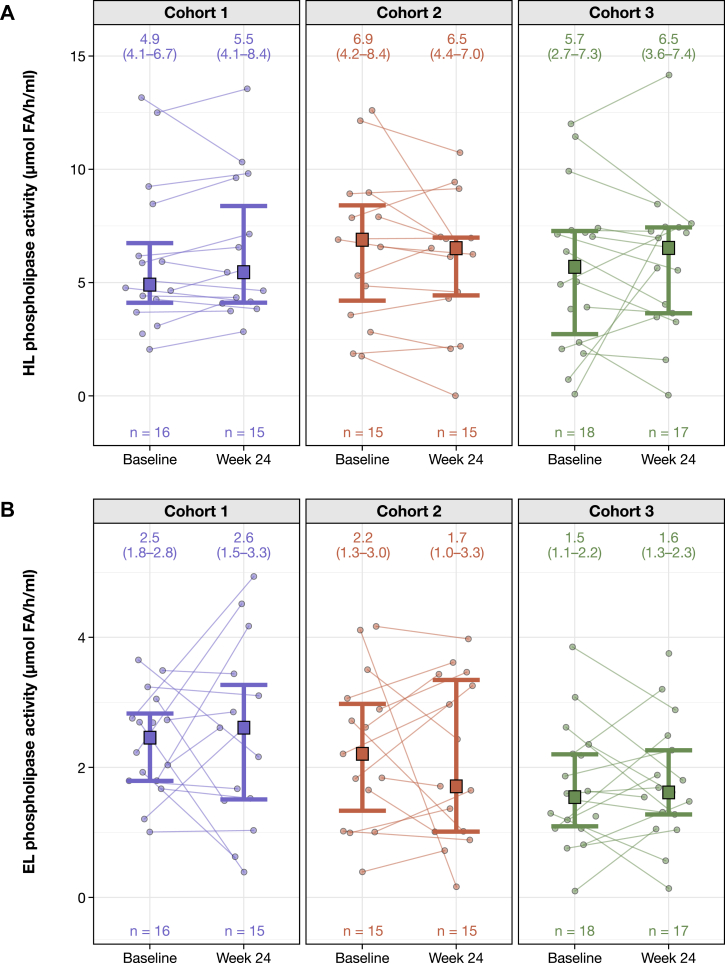
Assessment of (A) HL and (B) EL phospholipase activity via colorimetric assays shown for individuals and grouped according to cohort^a^. ^a^Bold lines indicate median (IQR) values. EL, endothelial lipase; FA, fatty acid; HL, hepatic lipase; IQR, interquartile range.

Assessment of HL and EL phospholipase activity measured via colorimetric assays for individuals and grouped according to cohort is shown in Figure 4. Neither HL nor EL phospholipase activity was significantly different between cohorts at baseline (Fig. 4; Supplemental Table S3 and S5). Neither HL nor EL phospholipase activity showed consistent changes following evinacumab treatment; there was high intra-patient variability (Fig. 4; Supplemental Table S5).

### Linear regression analysis of HL triglyceride lipase activity versus HL phospholipase activity

**Fig. 5:**
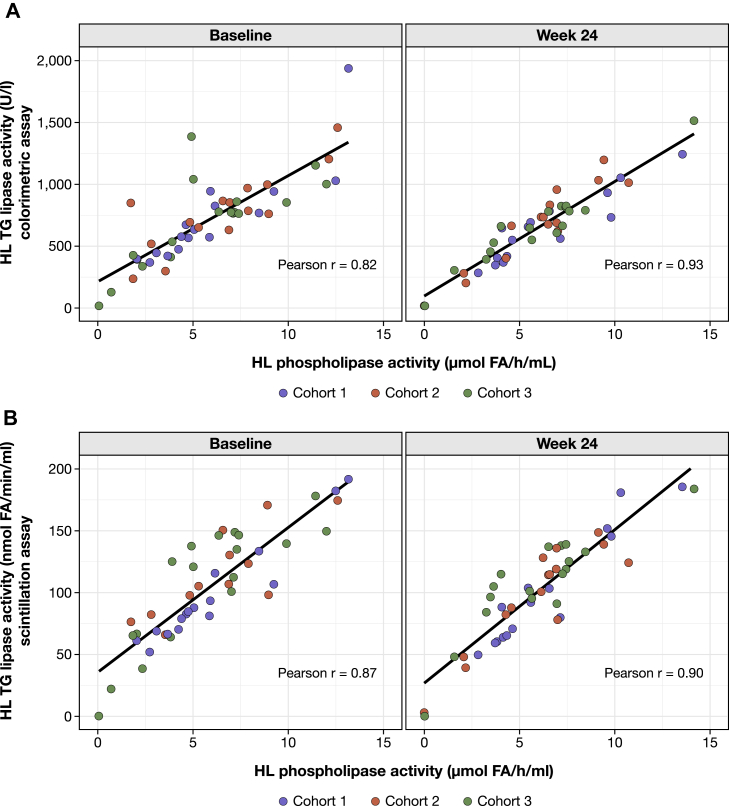
Scatterplots of HL phospholipase activity with HL triglyceride-lipase activity measured with (A) colorimetric and (B) scintillation assays at baseline and week 24. FA, fatty acid; HL, hepatic lipase; TG, triglyceride.

We performed a linear regression analysis of HL triglyceride lipase activity values measured with either colorimetric or scintillation assays versus HL phospholipase activity (Fig. 5). HL phospholipase activity was strongly correlated with HL triglyceride lipase activity measured by colorimetric assay at baseline (*r* = 0.82) and week 24 (*r* = 0.93), and HL triglyceride lipase activity measured by scintillation assay at baseline (*r* = 0.87) and week 24 (*r* = 0.90).

### Lipid parameters and correlation between changes in triglycerides and changes in lipase activity from baseline to week 24

The median (IQR) fasting triglycerides, total cholesterol, and apolipoprotein B (APOB) at baseline and week 24, along with the triglycerides/APOB and total cholesterol/APOB ratios at both time points, are presented in Supplemental Table S6. There was no correlation between the change from baseline to week 24 in triglycerides and the change in lipase activity (Supplemental Fig. S2).

## Discussion

Patients with sHTG who have experienced at least one episode of AP have a poor clinical prognosis, including being at high risk of recurrent events of acute pancreatitis and of chronic morbidity and mortality (24, 25). In a meta-analysis of 15 studies comparing sHTG-induced acute pancreatitis with non-sHTG-related acute pancreatitis, sHTG-related acute pancreatitis was associated with an increased odds ratio for shock (3.78), renal failure (3.18), respiratory failure (2.88), and mortality (1.90) (25).

LPL mediates the intravascular hydrolysis of triglycerides in chylomicrons and VLDL particles (21). As patients with LPL deficiency present with marked hypertriglyceridemia, accurate assessment of LPL triglyceride lipase activity is of clinical importance (21). Accordingly, this exploratory analysis investigated the association between LPL triglyceride lipase activity and genetic variants in patients with sHTG who received evinacumab as part of a phase 2 study (20). We systematically measured post-heparin LPL and HL triglyceride lipase activities using colorimetric and scintillation assays, as well as post-heparin HL and EL phospholipase activities using a colorimetric assay. Importantly, a linear regression analysis of HL triglyceride lipase activity showed high correlation between colorimetric and scintillation assay measurements. Furthermore, there was a high correlation between HL triglyceride lipase activity and HL phospholipase activity. This demonstrates the fundamental validity of our assay measurements. However, there was also high intra- and inter-patient variability for all lipase measurements. It is noteworthy that assay performance may vary among patients with different clinical backgrounds.

Baseline LPL triglyceride lipase activity, measured via colorimetric and scintillation assays (the latter as non-HL activity), was lower in cohort 1 (patients with FCS and bi-allelic LOF LPL pathway mutations) compared with cohort 2 (patients with MCS and heterozygous LOF LPL pathway mutations) and cohort 3 (patients with MCS without LPL pathway mutations). In contrast, HL triglyceride lipase activity was not different among the three cohorts. These findings align with our understanding of FCS and MCS and their varying effects on LPL activity, reinforcing the unreliability of the two conventional assays and supporting the use of chylomicronemia diagnosis-scoring systems (26).

Interestingly, we found evidence after evinacumab treatment for a modest increase in non-HL (mostly LPL) triglyceride lipase activity (assessed using the scintillation assay) at 24 weeks in cohort 1, which had the lowest LPL activity at baseline (most patients with biallelic LPL mutations have some low-level residual LPL activity). This is consistent with the mechanism of action of evinacumab inhibiting ANGPTL3, an inhibitor of LPL, and thus leads to activation of LPL (18). In contrast, there was no increase in HL triglyceride lipase activity, consistent with the fact that ANGPTL3 does not inhibit HL (19). Furthermore, although ANGPTL3 inhibits EL (18), we were unable to demonstrate an increase in EL phospholipase activity with evinacumab treatment. This may be due to the inherent limitations of the phospholipase activity assay.

Determination of LPL activity is complex. Commercially available assays to assess LPL activity typically use radiolabeled fluorogenic or chromogenic substrates (27). These are typically optimized to assess LPL activity following heparin administration to release LPL from heparan sulfate proteoglycans, which was employed in the two assays used here. However, this approach has certain disadvantages in a clinical setting, including the fact that patients must receive an intravenous injection of heparin. This prevents serial measurements of LPL, as LPL activity can only be assessed directly following the heparin injection (28). In addition, heparin also releases HL, which contributes to total lipolytic activity (28). Consequently, HL activity must be subtracted from total lipolytic activity to assess plasma LPL activity (28), as was done for the colorimetric and scintillation assays in this study. Further complicating the issue is that the pathophysiological relevance of post-heparin LPL activity is uncertain, as it is not clear to what extent heparan sulfate proteoglycan-bound LPL is active (28). Some alternatives to the post-heparin assessment of LPL activity have been developed. These include in vitro real-time fluorescence assays as well as approaches that enable direct assessment of VLDL-bound LPL-dependent VLDL-triglyceride hydrolysis through the measurement of non-esterified fatty acid release during in vitro incubations (27, 28).

In conclusion, this was an exploratory analysis of post-heparin triglyceride and phospholipase activities in three sHTG cohorts defined by LPL genotype at baseline and after 12 or 24 weeks of treatment with evinacumab. We found a trend towards lower baseline LPL (but not HL) triglyceride lipase activity in those with bi-allelic LPL pathway mutations, and an increase in this group in non-HL triglyceride activity (assessed using the scintillation assay) after receiving evinacumab. However, the observed inter-patient variability in LPL activity with both colorimetric and scintillation assays highlights the need for a standardized, robust, and reliable method to assess LPL activity.

## Relevant Patent Information

1) Methods and systems for biocellular marker detection and diagnosis using microfluidic profiling (PCT/US2019/026364) - Provisional.

2) Compositions and methods relating to the identification and treatment of immunothrombotic conditions (PCT/US2021/63104926) - Provisional.

## Data availability

Qualified researchers may request access to study documents (including the clinical study report, study protocol with any amendments, blank case report form, and statistical analysis plan) that support the methods and findings reported in this manuscript. Individual anonymized patient data will be considered for sharing: (1) once the product and indication has been approved by major health authorities (e.g., FDA, EMA, PMDA, etc.) or development of the product has been discontinued globally for all indications on or after April 2020 and there are no plans for future development; (2) if there is legal authority to share the data; and (3) there is not a reasonable likelihood of patient re-identification. Submit requests to https://vivli.org/.

## Supplemental data

This article contains supplemental data (29).

## Conflict of interest

The authors declare the following financial interests/personal relationships which may be considered as potential competing interests:

C. Vitali is currently employed at Arrowhead Pharmaceuticals. At the time of this study, she was employed at the University of Pennsylvania. P. Banerjee, R. Pordy, and P. Ehmann are employees of and stockholders in Regeneron Pharmaceuticals, Inc. R.S. Rosenson reports grants and/or personal fees outside the submitted work from Regeneron Pharmaceuticals, Inc., Amgen, Arrowhead Pharmaceuticals, Avilar Therapeutics, CRISPR Therapeutics, Editas Medicine, Eli Lilly, GlaxoSmithKline PLC, Intercept Pharmaceuticals, Life Extension, Lipigon, Kowa American Corporation, Meda Pharmaceuticals, New Amsterdam Pharma, Novartis, Organon, Precision BioSciences, Rona Therapeutics, UpToDate, Ultragenyx, Verve Therapeutics, and Viatris; and stock holdings in MediMergent, LLC. D.J. Rader reports consultancy fees/honoraria for scientific advisory board participation for Alnylam Pharmaceuticals, Novartis, Regeneron Pharmaceuticals, Inc., and Verve Therapeutics; and has ownership interest/partnership/principal in Vascular Strategies. Relevant patent information: 1) Methods and systems for biocellular marker detection and diagnosis using microfluidic profiling (PCT/US2019/026364) - Provisional 2) Compositions and methods relating to the identification and treatment of immunothrombotic conditions (PCT/US2021/63104926) - Provisional D.J. Rader reports consultancy fees/honoraria for scientific advisory board participation for Alnylam Pharmaceuticals, Novartis, Regeneron Pharmaceuticals, Inc., and Verve Therapeutics; and has ownership interest/partnership/principal in Vascular Strategies.
