# Supplementary material for: Post-heparin plasma lipase activities in patients with severe hypertriglyceridemia treated with evinacumab
Source: J Lipid Res. 2026 Mar 12;67(4):101020. doi: 10.1016/j.jlr.2026.101020 (PMC13091512; doi:10.1016/j.jlr.2026.101020)

# Supplemental Material

# Supplemental methods

## Inclusion criteria

Patients meeting the following criteria were included in the study:

- Males and females ≥18–75 years of age at screening.
- Previous documentation in the patient’s medical records of a fasting serum triglycerides (TGs) measurement ≥1000 mg/dl (11.3 mmol/l) on more than one occasion, and all fasting TG values >500 mg/dl (5.6 mmol/l) at screening.
- History of hospitalization and diagnosis of acute pancreatitis in the past 10 years. (the sponsor may elect to drop the time specification if sufficient enrollment does not occur within a reasonable period of time).
- On a stable lipid-modifying diet with or without medications (e.g., statins, niacin, omega-3 fatty acids). Lipid-modifying diet and doses of medications should be stable for at least 4 weeks (6 weeks for fibrates, 8 weeks for proprotein convertase subtilisin/kexin type 9 inhibitors) prior to screening.
- Body mass index of 18–40 kg/m^2^.
- Willing to consume no more than an average of two standard alcoholic drinks per day and a maximum of 15 standard alcoholic drinks per week for the duration of the study. (A standard alcoholic drink is the equivalent of 12 ounces (355 ml) of beer, 5 ounces (148 ml) of wine, or 1.5 ounces (44 ml) of hard liquor.)
- Willing to refrain from consumption of alcohol for 24 hours prior to each study visit.
- Willing to refrain from cigarette smoking for 24 hours prior to each study visit.
- Willing to consistently maintain previously recommended diet and exercise regimen for the duration of the study.
- Willing and able to comply with clinic visits and study-related procedures.
- Willing and able to provide signed informed consent.
- Able to understand and complete study-related questionnaires.

## Exclusion criteria

Patients meeting the following criteria were excluded from the study:

- A hospital or clinic discharge diagnosis of acute pancreatitis within 12 weeks of screening.
- Lipid apheresis or plasma exchange treatment within the last 4 weeks, or plans to undergo apheresis or plasma exchange during the time frame of the study.
- History of congestive heart failure, cerebrovascular accident, transient ischemic attack, unstable angina, coronary artery bypass graft, percutaneous coronary intervention, carotid surgery/stenting, or myocardial infarction within 3 months before the screening visit.
- History of bleeding disorders, esophageal varices, heparin-induced thrombocytopenia, or contraindications to receiving heparin.
- New clinically significant findings on 12-lead electrocardiogram that would place the patient at risk or interfere with participation in the study.
- Dose(s) of any permitted concomitant medications that have changed in the time period prior to screening.
- Presence of any clinically significant uncontrolled endocrine disease known to influence serum lipids or lipoproteins. (Note: Patients on thyroid replacement therapy are eligible if the dosage of thyroxine has been stable for at least 12 weeks prior to the screening visit.)
- Use of systemic corticosteroids, unless used as replacement therapy for pituitary/adrenal disease (in which case they must be a stable regimen for at least 6 weeks prior to the screening visit). (Note: Topical, intra-articular, nasal, inhaled, and ophthalmic steroid therapies are not considered as ‘systemic’ and are allowed.)
- Calculated creatinine clearance <45 ml/min (Cockcroft-Gault equation).
- History of drug or alcohol abuse within 1 year of screening.
- Exposure to another investigational drug or therapy within 30 days or within at least five half-lives (whichever is longer) prior to the screening visit.
- Blood donation of any volume within 1 month prior to the administration of the study drug
- Known hypersensitivity to monoclonal antibody therapeutics.
- History of malignancy within 5 years prior to screening (other than successfully treated nonmetastatic cutaneous squamous cell or basal cell carcinoma, and or localized carcinoma in situ of the cervix).
- Any medical or psychiatric condition that, in the opinion of the investigator, would place patient at risk, interfere with participation in the study, or interfere with the interpretation of the study results (e.g., cirrhosis or chronic active hepatitis, nephrotic syndrome, uncontrolled diabetes, uncontrolled hypertension).
- Known sensitivity to doxycycline or to any of the components of the investigational product formulation.
- Previous treatment with Glybera™ in the past 5 years.
- Positive serum human chorionic gonadotropin pregnancy test at the screening visit.
- Creatine phosphokinase >3 × the upper limit of normal (ULN) at the screening visit.
- Aspartate aminotransferase or alanine aminotransferase >3 × the ULN.
- Thyroid-stimulating hormone >1.5 × the ULN or below the lower limit of normal.
- Platelet count <75,000.
- Any patient who is an investigator or any sub-investigator, research assistant, study coordinator, or other staff directly involved in the conduct of the protocol, or a family member of staff involved in the conduct of the protocol.
- Pregnant or breastfeeding females.
- Females of childbearing potential* who are unwilling to practice a highly effective birth control method prior to the initial dose, during the study, and for 24 weeks after the last dose of the study drug. Highly effective contraceptive measures include:
  - Stable use of combined (estrogen- and progestogen-containing) hormonal contraception associated with inhibition of ovulation initiated two or more menstrual cycles prior to screening:
    - Oral.
    - Intravaginal.
    - Transdermal.
  - Stable use of progestogen-only hormonal contraception associated with inhibition of ovulation initiated two or more menstrual cycles prior to screening:
    - Oral.
    - Injectable.
    - Implantable.
  - Intrauterine device.
  - Intrauterine hormone-releasing system.
  - Bilateral tubal ligation
  - Vasectomized partner (Note: Vasectomized partner is a highly effective birth control method provided that the partner is the sole male sexual partner of the female of childbearing potential trial participant and that the vasectomized partner has received medical assessment of the procedure’s surgical success).
  - Sexual abstinence (Note: Sexual abstinence is considered a highly effective method only if defined as refraining from heterosexual intercourse during the entire period of risk associated with study treatments).

*Postmenopausal females must be amenorrheic for at least 12 months in order not to be considered of childbearing potential. Postmenopausal status will be confirmed by measurement of follicle-stimulating hormone. Pregnancy testing and contraception are not required for females with documented hysterectomy and/or oophorectomy.

## Exome sequencing and analysis

Briefly, 1 μg of genomic DNA was fragmented and prepared for exome capture with a custom reagent kit from Kapa Biosystems, Inc. (Wilmington, MA, USA). Samples were captured using the SeqCap VCRome 2.1 exome target design (Roche NimbleGen, Inc., Pleasanton, CA, USA) and sequenced using 75-bp paired-end sequencing on a HiSeq 2500 instrument (Illumina, San Diego, CA, USA) with v.4 chemistry. Following sequencing, data were processed using a cloud-based pipeline that used DNAnexus (Mountain View, CA, USA) and Amazon Web Services (Seattle, WA, USA) to run standard tools for sample-level data production and analysis. Sequence reads were mapped and aligned to the GRCh37/hg19 human genome reference assembly using Burrows-Wheeler Alignment–maximal exact matches (1). Single-nucleotide polymorphisms and insertion–deletion variants and genotypes were called using GATK’s HaplotypeCaller (Broad Institute, Cambridge, MA, USA). Standard quality-control filters were applied to called variants. Passing variants were classified, annotated, and analyzed using a Regeneron Genetics Center–implemented Mendelian analysis pipeline to evaluate their potential functional effects. Variants were annotated for their observed frequencies in population control databases, including the Single-Nucleotide Polymorphism database, the 1000 Genomes Project, the Exome Aggregation Consortium Database, and internal Regeneron Genetics Center databases, to filter out common polymorphisms and high frequency, likely benign variants. Algorithms for bioinformatic prediction of functional effects of variants (Likelihood Ratio Test, Polymorphism Phenotyping v2, Sorting Intolerant From Tolerant, Combined Annotation Dependent Depletion, and Mutation Taster), along with conservation scores, were incorporated as part of the annotation process of variants and used to inform on the potential deleteriousness of identified candidate variants. Individuals in this study were screened for variants in a list of 28 genes compiled for their reported associations with triglyceride or lipid levels.

# Reference

1. Li, H., and Durbin, R. (2009). Fast and accurate short read alignment with Burrows-Wheeler transform. *Bioinformatics* **25**: 1754–1760.

# Supplemental Table S1. Summary of patients by genotype.

| **n (%)** | **Cohort 1 (n = 17)** | **Cohort 2 (n = 15)** | **Cohort 3 (n = 19)** |
| --- | --- | --- | --- |
| Genotype categories for *LPL* |  |  |  |
| Homozygote | 8 (47.0) | 0 | 0 |
| Compound heterozygote | 5 (29.4) | 0 | 0 |
| Heterozygote | 0 | 8 (53.3) | 0 |
| Other genes |  |  |  |
| Homozygote |  |  |  |
| *APOA5* | 1 (5.9) | 0 | 0 |
| *APOC2* | 1 (5.9) | 0 | 0 |
| *GPIHBP1* | 1 (5.9) | 0 | 0 |
| Compound heterozygote |  |  |  |
| *LMF1* | 1 (5.9) | 0 | 0 |
| Heterozygote |  |  |  |
| *APOA5* | 0 | 3 (20.0) | 0 |
| *GPIHBP1* | 0 | 2 (10.0) | 0 |
| *LMF1* | 0 | 2 (10.0) | 0 |
| No LPL pathway mutations | 0 | 0 | 19 (100.0) |

Abbreviations: *APOA5*, apolipoprotein A5; *APOC2*, apolipoprotein C2; *GPIHBP1*, glycosylphosphatidylinositol anchored high density lipoprotein binding protein 1; *LMF1*, lipase maturation factor 1; *LPL*, lipoprotein lipase.

# Supplemental Table S2. Summary of triglyceride lipase activity assessed via colorimetric assays.

| **Median (IQR)** | **Cohort 1 (n = 17)^a^** | | **Cohort 2 (n = 15)** | | **Cohort 3 (n = 19)** | |
| --- | --- | --- | --- | --- | --- | --- |
|  | **Baseline** | **Week 24** | **Baseline** | **Week 24** | **Baseline** | **Week 24** |
| LPL, U/l | 117.0  (101.0 – 138.0) n = 16 | 84.0  (69.0 – 204.0) n = 15 | 192.0  (163.0 – 238.0) n = 15 | 169.0  (148.0 – 258.0) n = 14 | 242.0  (165.0 – 330.0) n = 18 | 213.0  (171.0 – 285.5) n = 16 |
| HL, U/l | 605.0  (467.0 – 853.5) n = 16 | 646.0  (412.0 – 757.0) n = 15 | 786.0  (641.0 – 916.5) n = 15 | 690.0  (512.0 – 894.0) n = 15 | 772.0  (419.0 – 900.0) n = 19 | 660.0  (528.0 – 790.0) n = 17 |

^a^One patient with a loss-of-function variant in *APOC2* was removed from the analysis.

Abbreviations: *APOC2*, apolipoprotein C2; HL, hepatic lipase; LPL, lipoprotein lipase; IQR, interquartile range.

# Supplemental Table S3. Summary of statistical analysis.

|  | **Baseline differences (ANOVA)** | **Change from baseline to  week 24 (paired *t*-test)** |
| --- | --- | --- |
| **Triglycerides** | | |
| LPL (U/l), colorimetric assay | | |
| Cohort 1 | *F*(2,46) = 5.50, *P* = 0.007 Cohorts 1 & 3 are different | *t*(14) = 0.04, *P* = 0.966 |
| Cohort 2 |  | *t*(13) = 0.16, *P* = 0.874 |
| Cohort 3 |  | *t*(14) = 1.55, *P* = 0.143 |
| HL (U/l), colorimetric assay | | |
| Cohort 1 | *F*(2,47) = 0.24, *P* = 0.786 No pairwise differences | *t*(14) = 1.51, *P* = 0.154 |
| Cohort 2 |  | *t*(14) = 1.56, *P* = 0.140 |
| Cohort 3 |  | *t*(16) = 0.66, *P* = 0.522 |
| Non-HL (nmol FA/min/ml), scintillation assay | | |
| Cohort 1 | *F*(2,43) = 3.24, *P* = 0.048 No pairwise differences | *t*(14) = -2.26, *P* = 0.040 |
| Cohort 2 |  | *t*(11) = -0.40, *P* = 0.696 |
| Cohort 3 |  | *t*(15) = -1.10, *P* = 0.289 |
| HL (nmol FA/min/ml), scintillation assay | | |
| Cohort 1 | *F*(2,43) = 0.57, *P* = 0.569 No pairwise differences | *t*(14) = 0.08, *P* = 0.941 |
| Cohort 2 |  | *t*(11) = -2.38, *P* = 0.037 |
| Cohort 3 |  | *t*(15) = -0.02, *P* = 0.983 |
| Total triglyceride lipase (nmol FA/min/ml), scintillation assay | | |
| Cohort 1 | *F*(2,43) = 1.57, *P* = 0.221 No pairwise differences | *t*(14) = -1.54, *P* = 0.145 |
| Cohort 2 |  | *t*(11) = 0.64, *P* = 0.533 |
| Cohort 3 |  | *t*(15) = -0.64, *P* = 0.530 |
| **Phospholipases** | | |
| EL phospholipase (µmol FA/h/ml), colorimetric assay | | |
| Cohort 1 | *F*(2,46) = 2.53, *P* = 0.091 No pairwise differences | *t*(14) = -0.33, *P* = 0.743 |
| Cohort 2 |  | *t*(14) = 0.46, *P* = 0.652 |
| Cohort 3 |  | *t*(15) = 0.21, *P* = 0.835 |
| HL phospholipase (µmol FA/h/ml), colorimetric assay | | |
| Cohort 1 | *F*(2,46) = 0.37, *P* = 0.694 No pairwise differences | *t*(14) = -0.58, *P* = 0.573 |
| Cohort 2 |  | *t*(14) = 1.45, *P* = 0.169 |
| Cohort 3 |  | *t*(15) = -0.31, *P* = 0.760 |

Abbreviations: ANOVA, analysis of variance; EL, endothelial lipase; F, F-statistic; FA, fatty acid; HL, hepatic lipase; LPL, lipoprotein lipase.

Supplemental Table S4. Summary of triglyceride lipase activity assessed via scintillation assays.

| **Median (IQR)** | **Cohort 1 (n = 17)^a^** | | **Cohort 2 (n = 15)** | | **Cohort 3 (n = 19)** | |
| --- | --- | --- | --- | --- | --- | --- |
|  | **Baseline** | **Week 24** | **Baseline** | **Week 24** | **Baseline** | **Week 24** |
| Non-HL, nmol FA/min/ml | 54.4  (35.5 – 62.4) n = 16 | 66.7  (53.0 – 95.0) n = 15 | 81.6  (60.0 – 103.9) n = 12 | 83.5  (62.4 – 108.2) n = 15 | 87.6  (47.1 – 106.5) n = 18 | 88.8  (76.1 – 103.7) n = 17 |
| HL, nmol FA/min/ml | 83.6  (69.9 – 109.0) n = 16 | 88.2  (64.6 – 124.7) n = 15 | 106.0  (93.9 – 135.4) n = 12 | 114.2  (80.1 – 126.1) n = 15 | 122.9  (65.6 – 144.6) n = 18 | 114.9  (95.4 – 132.9) n = 17 |
| Total, nmol FA/min/ml | 132.1  (112.2 – 174.8) n = 16 | 156.7  (114.6 – 240.0) n = 15 | 190.0  (147.2 – 229.6) n = 12 | 198.0  (159.7 – 226.4) n = 15 | 230.5  (127.5 – 246.9) n = 18 | 203.9  (183.1 – 241.8) n = 17 |

^a^One patient with a loss-of-function variant in *APOC2* was removed from analysis.

Abbreviations: *APOC2*, apolipoprotein C2; FA, fatty acid; HL, hepatic lipase; IQR, interquartile range.

Supplemental Table S5. Summary of phospholipase activity assessed via colorimetric assays.

| **Median (IQR)** | **Cohort 1 (n = 17)^a^** | | **Cohort 2 (n = 15)** | | **Cohort 3 (n = 19)** | |
| --- | --- | --- | --- | --- | --- | --- |
|  | **Baseline** | **Week 24** | **Baseline** | **Week 24** | **Baseline** | **Week 24** |
| EL, µmol FA/h/ml | 2.5  (1.8 – 2.8) n = 16 | 2.6  (1.5 – 3.3) n = 15 | 2.2  (1.3 – 3.0) n = 15 | 1.7  (1.0 – 3.3) n = 15 | 1.5  (1.1 – 2.2) n = 18 | 1.6  (1.3 – 2.3) n = 17 |
| HL, µmol FA/h/ml | 4.9  (4.1 – 6.7) n = 16 | 5.5  (4.1 – 8.4) n = 15 | 6.9  (4.2 – 8.4) n = 15 | 6.5  (4.4 – 7.0) n = 15 | 5.7  (2.7 – 7.3) n = 18 | 6.5  (3.6 – 7.4) n = 17 |

^a^One patient with a loss-of-function variant in *APOC2* was removed from analysis.

Abbreviations: *APOC2*, apolipoprotein C2; EL, endothelial lipase; FA, fatty acid; HL, hepatic lipase; IQR, interquartile range.

# Supplemental Table S6. Lipid parameters and their ratios at baseline and week 24.

| **Median (IQR), mg/dl** | **Cohort 1 (n = 17)^a^** | | **Cohort 2 (n = 15)** | | **Cohort 3 (n = 19)** | |
| --- | --- | --- | --- | --- | --- | --- |
|  | **Baseline** | **Week 24** | **Baseline** | **Week 24** | **Baseline** | **Week 24** |
| Fasting TG | 3223.7  (2723.7 – 3931.3) | 2307.0  (881.5 – 3629.5) | 1238.0  (943.7 – 3022.7) | 429.0  (306.5 – 761.5) | 1549.0  (1069.3 – 2189.5) | 1082.0  (187.0 – 1545.0) |
| Total cholesterol | 381.0  (284.0 – 412.0) | 205.0  (170.0 – 271.0) | 193.0  (180.5 – 240.5) | 137.0  (105.5 – 160.5) | 275.0  (196.5 – 371.5) | 203.0  (155.0 – 248.0) |
| APOB | 64.0  (56.0 – 90.0) | 60.0  (47.0 – 73.5) | 90.0  (77.0 – 101.0) | 85.0  (58.0 – 97.0) | 99.0  (79.0 – 125.5) | 91.0  (71.0 – 129.0) |
| **Ratios, median (IQR), mg/dl** |  |  |  |  |  |  |
| TG/APOB | 53.8  (41.2 – 63.0) | 42.4  (11.8 – 71.9) | 15.8  (9.7 – 30.0) | 6.5  (3.5 – 9.5) | 13.4  (10.5 – 20.6) | 8.6  (2.3 – 17.6) |
| Cholesterol/APOB | 5.5  (3.8 – 7.3) | 3.4  (2.0 – 5.9) | 2.3  (1.8 – 3.2) | 1.5  (1.4 – 1.9) | 2.6  (1.9 – 3.8) | 1.8  (1.7 – 2.2) |

^a^One patient with a loss-of-function variant in *APOC2* was removed from the analysis.

Abbreviations: APOB, apolipoprotein B; IQR, interquartile range; TG, triglycerides.

# Supplemental Fig. S1. Overview of study design.


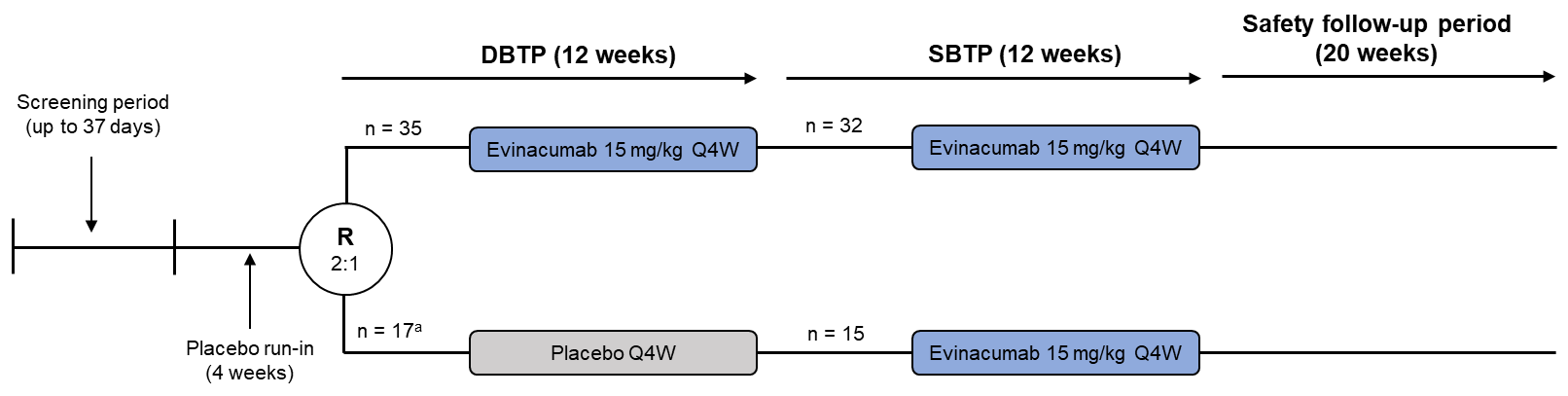


^a^One patient was randomized but not treated. Four patients did not complete the study due to withdrawal of consent.

DBTP, double-blind treatment period; Q4W, every 4 weeks; R, randomized; SBTP, single-blind treatment period.

# Supplemental Fig. S2. Correlation between changes in triglycerides and changes in lipase activity from baseline to week 24.


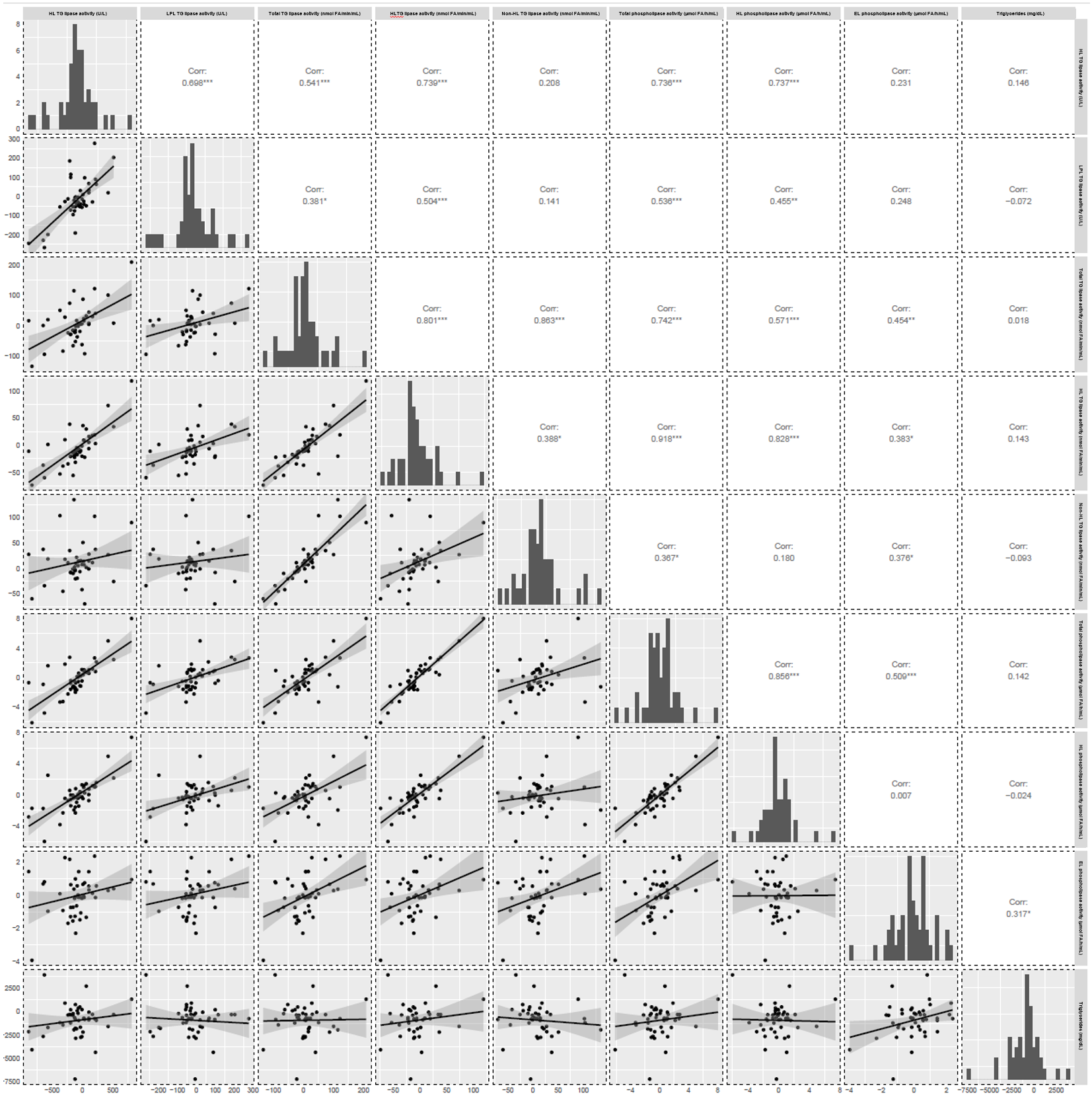

Supplement: Supplementary Figures and Tables [file mmc1.docx]
